# Iron deprivation enhances transcriptional responses to *in vitro* growth arrest of *Mycobacterium tuberculosis*

**DOI:** 10.3389/fmicb.2022.956602

**Published:** 2022-10-04

**Authors:** Sogol Alebouyeh, Jorge A. Cárdenas-Pestana, Lucia Vazquez, Rafael Prados-Rosales, Patricia Del Portillo, Joaquín Sanz, Maria Carmen Menéndez, Maria J. García

**Affiliations:** ^1^Department of Preventive Medicine and Public Health and Microbiology, School of Medicine, Autonomous University of Madrid, Madrid, Spain; ^2^Department of Theoretical Physics, University of Zaragoza, Zaragoza, Spain; ^3^Institute for Biocomputation and Physics of Complex Systems (BIFI), University of Zaragoza, Zaragoza, Spain; ^4^Corporación CorpoGen, Bogota, Colombia

**Keywords:** *Mycobacterium tuberculosis*, iron availability, transcriptomics, growth arrest, metabolic changes

## Abstract

The establishment of *Mycobacterium tuberculosis* (Mtb) long-term infection *in vivo* depends on several factors, one of which is the availability of key nutrients such as iron (Fe). The relation between Fe deprivation inside and outside the granuloma, and the capacity of Mtb to accumulate lipids and persist in the absence of growth is not well understood. In this context, current knowledge of how Mtb modifies its lipid composition in response to growth arrest, depending on iron availability, is scarce. To shed light on these matters, in this work we compare genome-wide transcriptomic and lipidomic profiles of Mtb at exponential and stationary growth phases using cultures with glycerol as a carbon source, in the presence or absence of iron. As a result, we found that transcriptomic responses to growth arrest, considered as the transition from exponential to stationary phase, are iron dependent for as many as 714 genes (iron-growth interaction contrast, FDR <0.05), and that, in a majority of these genes, iron deprivation enhances the magnitude of the transcriptional responses to growth arrest in either direction. On the one hand, genes whose upregulation upon growth arrest is enhanced by iron deprivation were enriched in functional terms related to homeostasis of ion metals, and responses to several stressful cues considered cardinal features of the intracellular environment. On the other hand, genes showing negative responses to growth arrest that are stronger in iron-poor medium were enriched in energy production processes (TCA cycle, NADH dehydrogenation and cellular respiration), and key controllers of ribosomal activity shut-down, such as the T/A system *maz*E6*/*F6. Despite of these findings, a main component of the cell envelope, lipid phthiocerol dimycocerosate (PDIM), was not detected in the stationary phase regardless of iron availability, suggesting that lipid changes during Mtb adaptation to non-dividing phenotypes appear to be iron-independent. Taken together, our results indicate that environmental iron levels act as a key modulator of the intensity of the transcriptional adaptations that take place in the bacterium upon its transition between dividing and dormant-like phenotypes *in vitro*.

## Introduction

Together with carbon sources, a key metabolic determinant for the survival and growth of *Mycobacterium tuberculosis* (Mtb) is iron. Iron starvation affects the main carbon metabolism pathways leading to downregulation of the metabolic activity, including glycolysis, oxidative phosphorylation and other core metabolic pathways (Kurthkoti et al., 2017). As such, iron availability is essential for the growth and pathogenicity of Mtb (Kolloli et al., 2019). Moreover, iron treatment of Mtb-infected mice impairs bactericidal activity of antibiotic drugs and promotes bacterial growth with the progression of the disease (Lounis et al., 2003). In addition, mutants of Mtb harboring deficiencies in genes associated with Fe-acquisition and transport are attenuated in macrophages and mouse models of infection (Madigan et al., 2015). In spite of these findings, an excess of iron could be toxic to the bacteria as well, due to the generation of toxic radicals (Padilla-Benavides et al., 2013; Rowland and Niederweis, 2013), highlighting the importance of a tight regulation of iron homeostasis for the survival of Mtb.

Since Mtb is an obligate pathogen with a complex infection cycle that involves both intracellular and extracellular phases, it has evolved different strategies to see its iron needs fulfilled at all stages of the infection cycle.

Initially, when the pathogen is phagocytosed by the macrophage, it encounters a hostile environment, characterized by the presence of a number of stressors (Schnappinger et al., 2003) that trigger its transition to a dormant state. Among these hostile features, the phagosomal environment offers low free iron levels to the bacterium, due to the presence of the Fe-sequestering host cell proteins such as transferrin, ferritin, and lactoferrin, with whom the pathogen must compete. Those sequestering proteins are also abundant in the necrotic center of the granuloma, making it, similarly to the intracellular niche, a low iron environment (Kurthkoti et al., 2017). Under these environmental constraints, Mtb activates a transcriptional program aimed at increasing and regulating iron acquisition, including the production of two different types of siderophores, such as mycobactin (MBT) and carboxymycobactin (c-MBT) (Madigan et al., 2015), as well as the machinery needed for their secretion (the ESX-3 type VII secretion system), and other molecules such as the MmpL4/S4 and MmpL5/S5 (mycobacterial membrane protein large), and the ABC transporter IrtA/IrtB in the inner membrane (Chao et al., 2019; Zhang et al., 2020). Upon acquisition, two proteins participate in the iron storage by Mtb, the bacterioferritin BfrA and the ferritin-like protein BfrB (Rodriguez, 2006).

When the bacilli are in the bloodstream, or growing extracellularly within the cavitary lesions characteristic for the active phase of the pulmonary form of TB disease, host’s heme is the major source of iron, contrary to the non-heme source route (Zhang et al., 2020). Under these circumstances, Mtb deploys a unique heme uptake program, including the heme-binding protein encoded by Rv0203, which hijacks the heme, in order to internalize it later. Two transcriptional repressors, namely *Zur* and *SmtB*, are also involved in the heme-dependent iron acquisition by Mtb (Zhang et al., 2020). These findings highlight bacterial strategies beside iron acquisition, which guaranty its homeostasis and indicate that such strategies may differ according to different environments during the infection cycle.

One of the main characteristics of Mtb is the abundance and complexity of the lipid content in its cell envelope (Brennan, 2003). Near half of the dry weight of the bacterial cell wall is composed of complex lipids, and a significant proportion of the genome capacity of the bacillus is related with the lipid metabolic activity (Cole et al., 1998). The lipid composition of Mtb ranges from essential compounds, such as mycolic acids, to disposable components that are often involved in modulating pathogenicity and virulence, either enhancing (thealose dimicolate, TDM) or suppressing (phthiocerol dimycocerosate, PDIM) the host immune responses (see Queiroz and Riley, 2017 for a revision). Similar to the different pathways involved in iron acquisition and homeostasis, distinct mycobacterial lipids are also differentially involved in the different phases of Mtb infection. For example, while TDM is important during the early stage of infection, being involved in the granuloma development; triacylglycerol (TAG) is abundant in the lipid droplets that Mtb develops while entering in a dormant state during latent infection (Santucci et al., 2016).

*In vitro*, the bacterial lipid composition also depends on the culture conditions, such as time of incubation or culture media composition (Guallar-Garrido et al., 2021). The lipid composition has a critical influence on the phenotype of the bacilli. For example, the loss of their characteristic acid-fast staining used in diagnostic is thought to be due to changes in the mycolic acid contents of the cell wall (Deb et al., 2009). Changes in the level of lipids during Mtb growth *in vitro* have been linked not only to changes in the expression level of the corresponding coding genes but also to post-transcriptional modifications, as it was suggested for the content of PDIM (Rens et al., 2021). Moreover, subculturing Mtb increases the proportion of PDIM-depleted bacteria (Domenech and Reed, 2009).

Iron is an important cofactor putatively related to lipid metabolism. Rodriguez et al. (2002) identified that the IdeR regulon was involved in lipid metabolism. Moreover, Madigan et al. (2015) found a relationship between iron deprivation and synthesis of phospholipids, establishing a link between iron availability and the lipid composition of Mtb. In addition, the content in TAG has been also described during *in vitro* growth of Mtb in iron-limited environment, both in the wild-type strain (Bacon et al., 2007) and in the *mbt*K mutant strain (Madigan et al., 2015). Beyond these findings, and even if iron is an important cofactor putatively related to lipid metabolism, little is known about the relation between iron depletion, growth profile, and lipid metabolism in Mtb.

Besides changes in lipid composition and iron homeostasis, Mtb bacteria undergo a broader series of phenotypic adaptations during the transition to dormancy *in vivo*. Many of these include the transcriptomic activation of defense mechanisms to the environmental stresses found by the pathogen upon phagocytosis (Schnappinger et al., 2003). These stresses include exposure to reactive oxygen and nitrogen species (Voskuil et al., 2011), deprivation of key nutrients including iron, hypoxia, and exposure to lipids such as cholesterol, whose metabolism by the bacteria imposes the need to control the pool of certain toxic intermediate metabolites, particularly propionyl-CoA (Lee et al., 2013). As a matter of fact, extensive literature exists on the development of sophisticated *in vitro* models of Mtb dormancy (reviewed in Gibson et al., 2018), based on the combination of several of those exposures during the culturing process. Even though some of these models have been highly instrumental in reproducing *in vitro* Mtb phenotypes that are largely comparable to what is found in dormant bacteria *in vivo* (Gibson et al., 2018), it is not clear whether bacteria sense these stresses independently or simultaneously. This is particularly true regarding iron, whose role as a signaling mediator of genome-wide responses leading to dormancy and resuscitation is only partially understood.

In the present work we have characterized the influence of iron availability in the transcriptional response and lipid content of Mtb, during the *in vitro* transition from exponential to stationary phase.

By doing so, we intend to offer a systematic view of the role of iron in modulating functional responses to growth arrest *in vitro*, as a proxy model to its transition from a replicative to a dormant phenotype. While iron availability effects on gene expression have been previously interrogated in the literature (Kurthkoti et al., 2017), a formal comparison of their relevance in shaping the transition from growing to slow-down dividing phenotypes is missing. Our results in this particular context indicate that, although the differences between lipid composition of Mtb from exponential and stationary cultures are largely independent of iron levels, Fe levels does exert a significant role in modulating transcriptional responses to growth arrest, when bacilli goes from active division at exponential to slow replication at stationary phase.

## Materials and methods

### Bacterial strains and culture conditions

*Mycobacterium tuberculosis* H37Rv was used in this study. As a first step, an inoculum from culture stored at −70°, was grown in Middlebrook 7H9 at 37°C. Then, adaptation to iron starvation was achieved by growing an inoculum in iron-depleted Minimal Media (MM), using glycerol at 1%, supplemented with AN (Albumin-NaCl) (MM-AN) as published by Rodriguez et al. (2002). Bacteria were considered adapted to that iron-depleted environment after rising three sequential pre-cultures at optical density (OD) of 1.5–2.0, conditions that allowed bacterial growth in a complete iron depleted environment (Rodriguez et al., 2002). Bacterial manipulations were made in a BSL3 laboratory.

Those adapted bacilli were used to start cultures supplemented with iron (+Fe, 50 μM) and without iron (−Fe). Cultures in MM-AN (+Fe and −Fe) were performed in 250 ml bottles with 50 ml of fresh media each, at an initial OD of 0.03–0.05, using a roller circle at 4 rpm. Cultures were collected at exponential phase (OD approximately 0.5) and stationary phase (OD > 2). Bacteria were harvested by centrifugation at 6,500 rpm. Two biological replicates per condition were processed.

After being collected, bacteria were analyzed by acid-fast double staining Auramine/Red Nile (Deb et al., 2009). Samples were protected with a coverslip using Vectashield™ as a mountain medium and examined in a Nikon Eclipse *T*i fluorescence microscope. Colony forming units (CFUs) were also determined, in each of the conditions, by performing 10-fold serial dilutions up to a 10^–6^ and plated onto Middlebrook 7H10 agar. Colonies were counted along 2 weeks of plates incubation at 37°C. Bacterial sediment for RNA isolation was resuspended in 0.1–0.2 g/ml of guanidinium chloride (GuClH) and stored at −70°C until RNA purification. Bacterial sediment for total lipids isolation was washed twice in PBS pH7 1x-Tyloxapol 0.05%, distributed in two aliquots for mycolic acids and total lipids, respectively. The final sediments were stored at −70° until processing for lipid extraction.

### RNA isolation and purification

Samples were centrifuged and 1 volume (V) of GuClH was mixed with 1 V of Tr-reagent Kit Direct-zol RNA Miniprep Plus (Zymo Research) in a tube containing glass beads (150–212 mm; Sigma, St Louis, MO, USA). The tubes were mixed softly, and mechanically lysed with FastPrep (Bio101 Savant), using seven pulses of 50 seconds at 5 m/s speed, with 5 min on ice after each pulse. The mixture was centrifuged at 12,000 rpm for 5 min and the supernatant was collected for isolation of RNA, according to the manufacturer’s instructions, adding DNAse treatment in the same process. The pellet was resuspended with 100 μl H_2_O pre-treated with diethyl pyrocarbonate (H_2_O-DEPC). Efficacy and purification of RNA were tested by 2100 Bioanalyzer (Agilent Technologies). Supplementary Table 1 shows the purification data of the eight RNA samples.

### Lipid isolation and analysis

Isolation of mycolic acids was performed following previously described procedure (Dupont and Kremer, 2014). In brief, pellets were resuspended in 2 ml of tetrabutylammonium hydroxide (TBAH) and incubated at 100°C overnight. Esterification of mycolic acids was started by adding 4 ml CH_2_Cl_2_, 300 μl CH_3_I and 2 ml of H_2_O for 1 h at room temperature. The mixture was centrifuged at room temperature to allow phase separation. The upper phase was discarded; the remaining organic phase was washed with H_2_O and centrifuged, discarding the upper phase; this step was repeated three times. The lower phase was dried, 3 ml of diethyl ether was added to the residue and sonicated in a water bath at room temperature. The mixture was centrifuged and transferred to a pre-weighted glass tube; finally, the diethyl ether was evaporated, and the remaining residue was resuspended in CH_2_Cl_2_ to final concentration, according to residue weight (Supplementary Table 2).

Isolation of total lipids was performed according to previously described procedure (Guallar-Garrido et al., 2021). In summary, chloroform:methanol (1:2 v/v) solution was added to 0.2 g pellet, and incubated in constant stirring overnight. The following day it was filtered through a filter paper and collected into a new glass tube. The liquid phase was dried and stored at −20°C. To the remaining residue, chloroform:methanol (2:1 v/v) solution was added and incubated in constant stirring overnight. After resting for at least 1 h, the clear phase was transferred to the same glass tubes containing lipids, the organic solvent containing lipid was evaporated, and the remaining residue was resuspended in chloroform to final concentration, according to the residue weight. Supplementary Table 2 shows the purification data of lipids in the 8 samples.

Thin layer chromatography (TLC) analysis: for each condition, an equal concentration of lipids was loaded on the silica-TLC plate, inserted into, and run a saturated TLC chamber containing the mobile phase. Two-dimensional TLC analysis of phosphatidyl-inositol mannosides (PIMs) was performed in two directions: (60:30:6) chloroform:methanol:water and (40:25:3:6) chloroform:acetic acid:methanol:water, respectively.

The plates were removed and revealed by spraying with 10% molybdatophosphoric acid hydrate in ethanol followed by heating the plate at 120°C (Guallar-Garrido et al., 2021).

### RNA-seq data collection and pre-processing

Purified RNA samples were sent to Macrogen, Inc. (Seoul, South Korea) for a complete set of tasks: quality control, library preparation, sequencing, and mapping. Libraries were prepared using the kit TruSeq Stranded Total RNA (NEB Microbe) and the libraries were sequenced using an Illumina Novaseq. Phred scores in the raw fastq files produced were above 30 in more than 95% of bases sequenced (95.5–96.1% of Q30 bases). Adapter sequences and low-quality read ends were trimmed using trimmomatic (v. 0.38, Bolger et al., 2014). Trimmed reads were aligned to reference genome (*M. tuberculosis* H37Rv, ASM19595v2) with Bowtie (v. 1.1.2, Langmead et al., 2009), and vectors of reading counts per gene were compiled using HTseq (v. 0.10.0, Anders et al., 2015). The whole sequencing process was performed twice (two technical replicates) for each of the two biological replicates in each condition. Statistical analysis showed technical variability was negligible compared to biological variability across biological replicates within the same condition. This leads us to consider the first set of biological replicates alone, neglecting the inclusion of technical replicates in downstream analyses, as the most conservative choice in order to avoid the artificial inflation of the statistical significance of our results. Supplementary Table 3 summarized the level of expression determined by RNA-seq.

All sequences obtained from RNAseq experiments are available at the GEO accession number: GSE213943.

### Differential expression analyses

All statistical analyses were conducted using R programming language (R Core Team, 2021). Principal component analysis was done on log-counts per million data, normalized across samples using Trimmed-M-means algorithm, as implemented in R package edgeR (Campbell et al., 2007). Differential expression analyses were conducted using DEseq2 (v. 1.28.1, Love et al., 2014).

**FIGURE 1:**
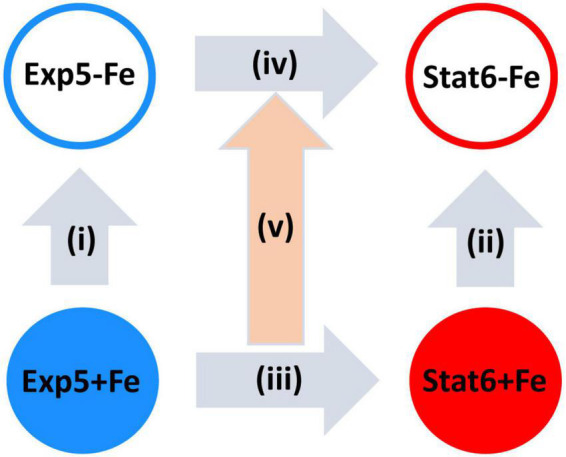
Schematic representation of the comparisons made. Vertical arrows represent the effect of iron deprivation at exponential [Exp5 (i)] and at stationary [Stat6 (ii)] phases of growth. Horizontal arrows represent the effect of transition from exponential (Exp5) to stationary (Stat6) phases of growth with (iii) and without (iv) iron. The central arrow represents the second order contrast on the interaction of growth arrest with iron availability (v). Exp5+Fe, exponential culture with iron; Exp5–Fe, exponential culture without iron; Stat6+Fe, stationary culture with iron; Stat6–Fe, stationary culture without iron.

Four primary statistical contrasts were interrogated according to the design sketched in Figure 1. These included iron deprivation effects at exponential (contrast i) and stationary phases (contrast ii) as well as growth arrest effects, that is, differences in expression between stationary ad exponential, evaluated either, iron rich (contrast iii), or iron deprived cultures (contrast iv). A fifth, second order contrast was also estimated: the interaction between iron availability and growth arrest effects (i.e. the impact of iron on responses to growth arrest). As described in Love et al. (2014) read count data was modeled using the negative binomial distribution. The statistical significance of the log_2_FCs associated to each contrast was assessed through a Wald test using shrunken dispersion estimates. Differential expression analyses were conducted using DEseq2 (Love et al., 2014). Then, *p*-values were corrected for multiple testing using the Benjamini–Hochberg method (Benjamini and Hochberg, 1995), and effect sizes were also shrunken using DESeq2 function lfcShrink. Genes showing FDR <0.01 were considered differentially expressed (DE) for first-order contrasts i–iv, while a threshold of FDR <0.05 was used for the interaction.

## Results

In order to understand the influence of iron in the growth of Mtb in a medium with glycerol as main carbon source, bacteria were adapted to an iron-depleted media and then grown in iron-free (−Fe) and iron-enriched (+Fe) media, allowed to grow up to exponential (Exp5) or stationary (Stat6) phases.

Table 1 summarizes the general data derived from those cultures. Despite the differences in OD, the number of CFUs was similar comparing Exp5 to Stat6 phases of growth, indicating the expected decreasing proportion of viable bacilli once the culture enters into stationary phase.

**TABLE 1:** Culture conditions and CFUs data.

Cultures	Incubation	OD		CFUs/ml
		Initial	Final	
Exp5−Fe (1)	3d	0.03	0.49	6.4E+4
Exp5+Fe (1)	3d	0.03	0.55	4.4E+4
Stat6−Fe (1)	14d	0.05	2.8	1.3E+3
Stat6+Fe (1)	14d	0.05	2.95	2.6E+4

Two biological replicates of each condition were performed. Number of the replica is indicated between brackets. Exp5, exponential culture; Stat6, stationary culture; −Fe, cultures without iron; +Fe, cultures with iron.

**FIGURE 2:**
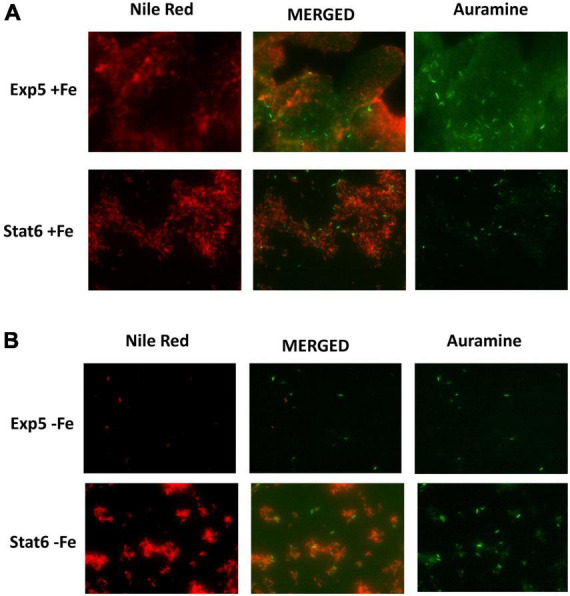
Double staining Auramine/Nile Red at exponential and stationary culture phases. Same frame of cultures with **(A)** and without **(B)** iron at exponential (Exp5–Fe) and stationary (Stat6–Fe) phases of growth stained with Auramine (right) and Nile Red (left). Corresponding merged image is also shown in the central panel.

Microscopy of double stained Auramin/Red Nile smears was used to characterize the cultures according to bacterial status. Extensions of cultures are shown in Figure 2. As expected, a low number of bacilli was seen in exponential culture compared to stationary, with bacilli showing stronger clumped phenotype at this last phase of growth. Moreover, red bacilli, showing inner lipid content in the cells, were identified at exponential and stationary phases irrespective of the iron content. However, they were in a higher proportion in the latter condition. The lipid content detected has been related with the accumulation of TAGs by the bacteria (Garton et al., 2008).

### Global transcriptomic growth arrest remodels the transcriptome of *Mycobacterium tuberculosis* analyses

As sketched in Figure 1, we have processed two biological replicates in four experimental conditions, extracted RNA from the resulting eight samples and conducted RNA-seq.

Table 2 shows the general overview of the RNAseq results. Although the total reads were similar among the four conditions tested, an increase of reads corresponding to non-coding sequences (CDS) was detected in the stationary phase, compared to exponential, irrespective of the iron availability in the cultures. High expression of non-CDS in the stationary phase of growth of Mtb has been reported by our and other groups (Rodríguez et al., 2014; Aguilar-Ayala et al., 2017; Del Portillo et al., 2019) and was previously explained by the increased expression of non-coding RNAs (ncRNAs). This explanation also applies to our setting, where percentages of ncRNAs, within stable RNAs reads, were about six times higher in Stat6 compared to Exp5 cultures. As expected, percentages of rRNAs were higher in Exp5 compared to Stat6. On the contrary, both tRNAs and miscellaneous RNAs remained at similar levels in the four conditions tested.

**TABLE 2:** General description of the transcriptomic results.

Number (%) of reads in the following conditions				
Reads	Exp5−Fe	Exp5+Fe	Stat6−Fe	Stat6+Fe
	
	(%) all	(%) all	(%) all	(%) all
All	15.98	14.8	13.0	12.3
CDS	12.6 (78.9)	11.6 (78.1)	1.7 (13.1)	2.7 (21.7)
stable RNAs	3.4 (21.1)	3.2 (21.9)	11.3 (86.9)	9.6 (78.3)

	**% stable RNAs**	**% stable RNAs**	**% stable RNAs**	**% stable RNAs**

ncRNAs	21.5 (6.4)	0.2 (5.2)	4.1 (36.6)	3.3 (34.1)
tRNAs	0.5 (0.1)	0.0041 (0.1)	0.0009 (0.01)	0.001 (0.01)
rRNAs	2.4 (70.7)	2.4 (73.5)	4.6 (41.1)	4.0 (41.9)
Misc RNAs	0.8 (22.8)	0.7 (21.1)	2.5 (22.2)	2.3 (23.9)

Total number (in million) of reads determined under the different conditions; and number (percentages) of reads respect to all reads (upper part) and respect to stable RNAs (lower part). Mean value of two replicates was presented. CDS, coding sequence; ncRNAs, non-coding RNAs; tRNAs, transfer RNAs; rRNAs, ribosomal RNAs; misc RNAs, genes *rnp*B and *ssr*.

**FIGURE 3:**
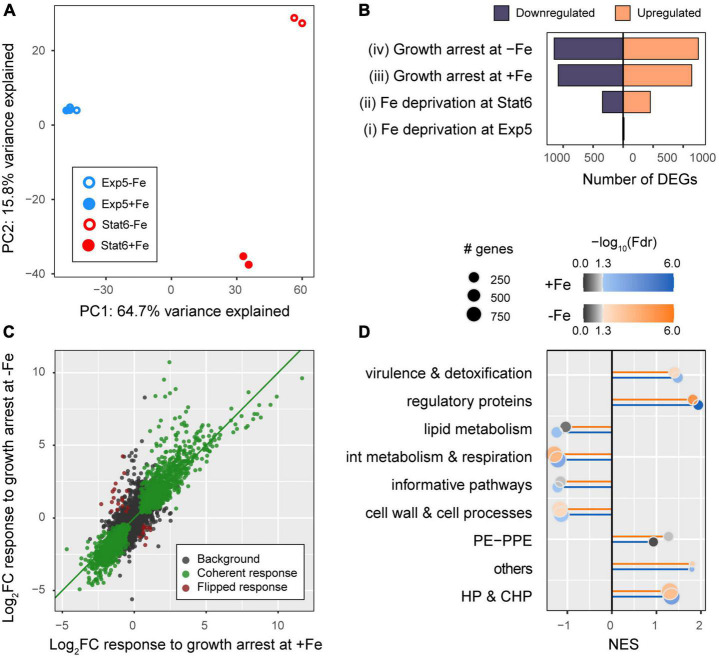
Overall description of the dataset. **(A)** Principal component analyses (PCA) of log-counts per millions normalized across samples (see “Materials and methods”): PC1-growth phase; PC2-iron availability. **(B)** Number of DE genes that were upregulated (orange) or downregulated (blue) in the four comparisons made: iron deprivation at exponential phase Exp5 (contrast i) and at stationary phase Stat6 (contrast ii) as well as growth arrest (transition from Exp5 to Stat6) with iron (contrast iii), and without iron (contrast iv). See Figure 1 for further explanation. **(C)** Effect sizes of the changes in gene expression in response to growth arrest at +Fe (*x*-axis) versus –Fe cultures (*y*-axis). Highlighted in green and maroon colors, we show genes that were DE in both conditions (at FDR <0.01), whose responses to growth arrest in –Fe and +Fe were directionally coherent (green), or flipped (maroon), and no DE (black). **(D)** Gene set enrichment analyses of genes annotated within Tuberculist functional categories.

After removing of genes poorly expressed [mean (fpkm) <2 in all 4 conditions] a total of 3,899 genes were selected for downstream analyses (Supplementary Table 3). As expected, principal component analyses (PCA) points to the existence of pervasive effects of growth arrest on gene expression since growth phase appears strongly aligned to the first principal component of the gene expression data (Figure 3A, PC1-growth phase Pearson correlation: *r* = 0.98, *p* = 1.2E−5), explaining as much as 64.7% of the total variance in the data. In turn, the second principal component, explaining 15.8% of the total variance, captures the effects of iron deprivation exclusively at the stationary phase (PC2-iron Pearson correlation: *r* = 0.99, *p* = 4.3E−4, from Stat6 samples alone). Finally, no principal component appears correlated to iron deprivation effects at Exp5, coherent with the comparatively weaker statistical evidence found for iron deprivation effects at the exponential phase than at the stationary phase (Figure 3A).

To further delineate these global patterns, we conducted differential expression analyses using DEseq2 (Love et al., 2014). While iron deprivation effects on gene expression were less common than growth arrest effects (Figure 3B), growth status was found to impact as many as 2,227 and 2,401 genes, in +Fe or −Fe media, respectively (Supplementary Figure 1). Among these genes, 1,903 (85.5%) are shared, representing an enrichment odds ratio OR = 13.8 (one-tailed Fisher exact test *p* < 2.2E−16). In agreement with this result, the effect sizes of the responses to growth arrest are largely independent of iron availability, with an overall, genome-wide, correlation of *r* = 0.878, *p* < 2.2E−16. Among those 1,903 genes with shared growth arrest effects, as many as 1,874 (98.5%) were coherent in sign (colored green in Figure 3C) in −Fe and +Fe cultures. Furthermore, functional enrichments across growth arrest effect size ranks in Tuberculist categories are strongly correlated between +Fe and −Fe cultures (Figure 3D). We also compared the distributions of log-fold changes observed in protein coding and ncRNAs in each condition. As seen in Supplementary Figure 2, we found that ncRNAs, unlike protein coding genes, tended to be systematically upregulated upon growth arrest, independent of iron availability.

### Iron deprivation enhances transcriptional responses to growth arrest

While the results described above indicate that responses to growth arrest are *qualitatively* independent from iron, both in the significance and the direction of a majority of the genes under analysis, we then interrogated for the presence of *quantitative* effects of iron deprivation on the size of the responses to growth arrest, namely, iron-growth interactions (contrast v, Figure 1).

From a straight comparison between the number of DE genes as a function of iron availability, either in Exp5 and Stat6 phases, we see that iron impacts gene expression of more than 20% of tested genes in Stat6 phase (797 genes, FDR <0.01) (Supplementary Table 4), while the evidence of iron deprivation effects at exponential phase is remarkably weaker, as we only observe statistically significant effects in 10 genes (that is, less than 0.3% of the genes tested genome wide). Importantly, these 10 genes are far from being randomly distributed genome-wide, as all but 2 of these genes are members of the mycobactin operon, coding for enzymes involved in mycobactin biosynthesis (*mbt*B, C, D, E, F, G, H, and I), indicating that half of the operon was upregulated in that condition. Furthermore, the entire *mbt* operon, with the exception of *mbt*J, was also significantly upregulated upon iron deprivation in the stationary phase, and iron effects on all genes were larger in the latter than in the former phase (Supplementary Figure 3).

Therefore, the asymmetric role of iron availability in modulating gene expression after, but not before growth arrest, directly implies the existence of significant iron-growth interactions, which we were able to ascertain statistically for 714 genes at 5% FDR.

**FIGURE 4:**
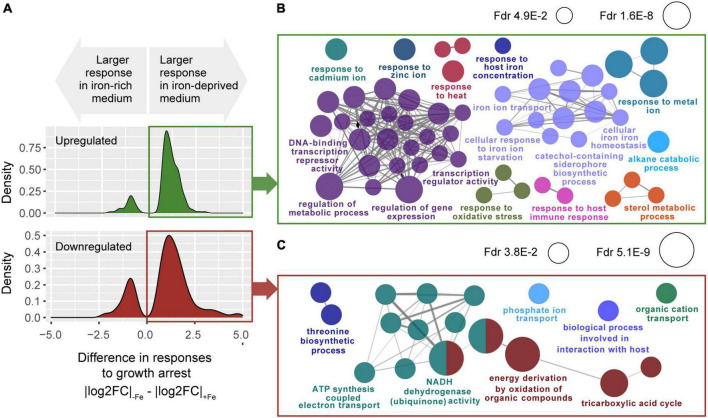
Transcriptomic response in the interaction of growth arrest with iron deprivation. **(A)** Distributions of the differences between absolute responses to growth arrest in –Fe versus +Fe cultures, for genes showing significant iron-growth interactions. Top: genes up-regulated upon growth arrest in both iron-rich medium and iron-deprived medium cultures, with a significant growth-iron interaction at FDR <0.05 [*n* = 326, of which 254 (77.9%) were more strongly upregulated in –Fe than +Fe cultures]. Bottom: genes downregulated upon growth arrest in both iron-rich medium and iron-deprived medium cultures, with a significant growth-iron interaction FDR <0.05 [*n* = 202, of which 175 (86.6%) were more downregulated in –Fe than +Fe cultures]. **(B)** Gene ontology terms enriched among the *n* = 254 genes more upregulated (EU genes) in –Fe cultures than in +Fe (one-tailed hypergeometric test, FDR <0.05). **(C)** Gene ontology terms enriched among the *n* = 175 genes more downregulated (ED genes) in –Fe cultures than in +Fe (one-tailed hypergeometric test, FDR <0.05).

Among those 714 genes, we found 528 whose response to growth arrest, still dependent on iron availability in its size, has the same direction regardless iron. In these genes, the effect of iron on the response to growth is merely quantitative, which renders them specially interesting and functionally interpretable. As many as 81.2% of those genes (429 over 528) showed increased responses to growth arrest under iron deprivation (Figure 4A, genes with |*_log_FC*|_−*Fe*_ − |*_log_FC*|_+*Fe*_ > 0) implying that, genome-wide, lack of iron primes the bacterium to deploy a more marked transcriptomic adaptation to growth arrest. Those genes were distributed between 254 genes that were more upregulated in response to growth arrest in −Fe cultures [which we will refer to as “enhanced upregulated” (EU) genes], and 175 genes that were more repressed [henceforth referred to as “enhanced downregulated” (ED) genes] (Supplementary Tables 4, 5 and Supplementary Figure 4). Next, we used Cytoscape plugin ClueGO (Bindea et al., 2009; Korotkevich et al., 2021) to interrogate for biological process enriched among these genes. EU genes (*n* = 254) appear enriched in responses to iron ion starvation, metal homeostasis and regulatory functions (Figure 4B and Supplementary Table 5). Notably, ED genes were enriched in functions related to central carbon source metabolic pathways, such as tricarboxylic acids (TCA) cycle and electron transport (Figure 4C and Supplementary Table 5).

Transition to dormancy is frequently associated with an adaptive response to hostile environments, exemplified by the intracellular environment found by the bacteria when it enters the phagosome, that leans on two major components in what regards transcriptional adaptation: activation of regulatory programs and activation of pathways to slow down metabolism and replication (Schnappinger et al., 2003). In the following sections, we analyze the genes present and pathways enriched among EU and ED genes, that appear to be primarily involved in these processes.

### Iron deprivation enhances transcriptional activation of genes involved in responses to intracellular host-related stresses (enhanced upregulated genes)

**FIGURE 5:**
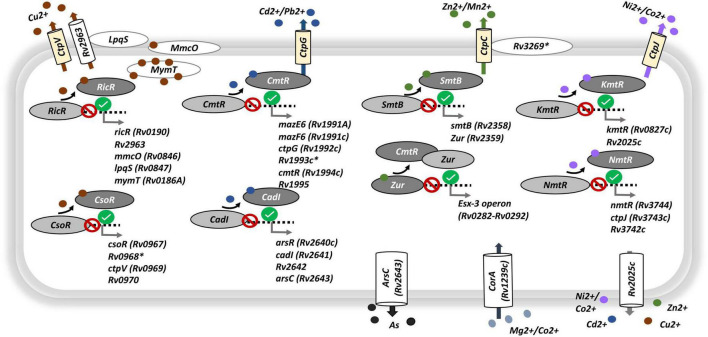
Genes associated to metal transport and regulation. Circular red and green signals indicate, respectively, the repression or activation of the corresponding regulatory gene-target in response to growth arrest being enhanced by iron deprivation [interaction, contrast (v)]. Light yellow barrel boxes, cation-transport ATPases type-P; white barrel and oval boxes, transmembrane proteins, membrane proteins or ions canals; light oval gray, repressor linked to related promoter region; dark oval gray, metal-repressor complex separated from related promoter region; small dense circles, different metals, each metal was represented by different color. Black: upregulated responses to interaction (EU genes). See Supplementary Table 4 for gene expression levels.

Among the ontology terms found to be enriched among the genes whose up-regulation upon growth arrest is enhanced by iron deprivation (EU genes) we found a series of terms related to the usage, homeostasis, and transport of assorted metal ions. As summarized in Figure 5, where we include the main genes contributing to the enrichments shown in Figure 4B, these include metal regulators and sensors, such as: *cso*R and *ric*R (for cupper); *cmt*R and *cad*l (for cadmium); *smt*B and *zur* (for zinc); and *kmt*R and *nmt*R (for nickel/cobalt). These regulatory proteins act as repressors when its surrogate metal is limited, and, except for *zur*, they are excised from their promoter-regulon region when linked to the metal. The release of those regulators from the promoter regions activates the transcription of their corresponding regulons, which includes those genes required for the metal turn-over. This is exemplified by genes Rv2963, *mmc*O, *lpq*S and *mym*T, all members of the *ric*R regulon (Figure 5 and Supplementary Tables 4, 5). Furthermore, the response of regulators was paired with secretion of transport proteins for each of those metals, such as cation-transport ATPases (*ctp*C, *ctp*G, *ctp*J, and *ctp*V) (Figure 5). These results emphasize the requirement of the bacteria not only for use but also for detoxification of metal ions in the cytoplasm, a metabolic activity crucial to maintaining their homeostasis.

Concerning iron homeostasis our results agreed with those previously described (Rodriguez et al., 2002; Rodriguez, 2006; Kurthkoti et al., 2017) and are summarized in Supplementary Figure 5. The pivotal regulator that allows iron homeostasis is IdeR. IdeR induces the expression of genes involved in iron uptake and storage, such as those coding for mycobactins, and bacterioferritins (Gold et al., 2001; Choudhury et al., 2021). None of those were DE in response to growth arrest in high iron medium. Conversely, *bfr*A was an EU gene and *bfr*B was ED gene in response to growth arrest in iron deprivation. We also identified differences in iron-transport proteins. The iron transport proteins *irt*A and *irt*B genes, coding for iron-ABC transporter were EU genes, and other membrane transporters genes, such as *mmp*L4/S4 and *mmp*L5/S5 were also differently used according to the iron availability (Supplementary Table 4).

These results indicate that absence of iron serves to the bacterium as a cue to enhance, upon growth arrest, the activation of genes involved in the metabolism of other metals in addition to iron, whose homeostasis is expected to become challenging for the pathogen in the intracellular milieu. Similarly, among the GO terms enriched in EU genes, we found processes related to the responses of stress cues that are considered cardinal features of the phagosomal environment and the host-pathogen cross-talk that happens there. These include responses to oxidative stress, response to host immune responses, and sterol metabolism, necessary for the bacterium to adapt to the high levels of cholesterol present in the host (Singh et al., 2009; Figure 4B and Supplementary Tables 4, 5).

### Iron deprivation leads to a stronger repression of energy production pathways and ribosomal activity (enhanced downregulated genes)

As shown in Figure 4C, the genes whose downregulation upon growth arrest was enhanced by iron deprivation (ED genes) are enriched among biological processes primarily related to basic carbon usage such as the TCA cycle and energy production such as the oxidative phosphorylation.

**FIGURE 6:**
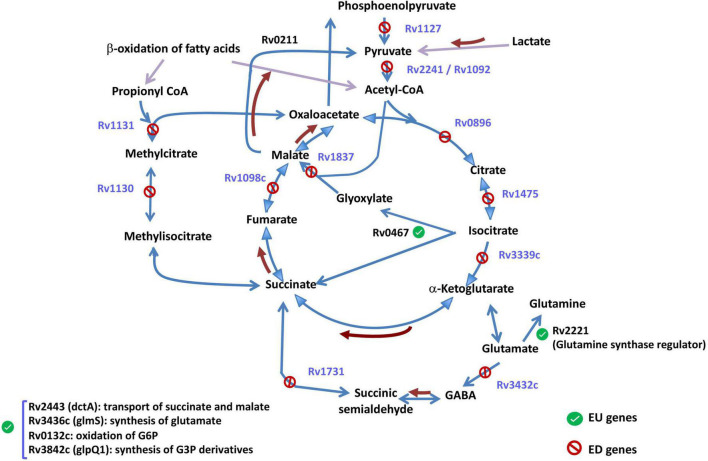
Genes within the TCA cycle and related metabolic pathways. In blue, enhanced-downregulated genes (ED) (whose abrogation upon growth arrest is inhanced by iron), and in black, enhanced-upregulated genes (EU). Brown arrows identify reactions whose genes were no differentially expressed. See Supplementary Table 4 for gene expression levels.

As summarized in Figure 6, half of the genes involved in the TCA cycle were ED genes, implying that completion of the cycle at the alpha-ketoglutarate and malate steps is more strongly shut down upon growth arrest when iron is unavailable. Moreover, the response to growth arrest of the glyoxylate shunt was also modified by iron. Although the main gene of this shunt (*icl*) was more strongly upregulated by growth arrest in −Fe medium (EU gene), the malate synthase G, controlling the next step from glyoxylate to malate is an ED gene (Supplementary Table 4). Interestingly, the gene Rv2349, a possible TetR-family transcriptional regulatory gene, found that putatively control the expression of *icl* (Minch et al., 2015), is also an EU gene (Supplementary Table 4).

The strong abrogation observed for the TCA cycle and adjacent pathways poses the question of what would be the escape route that bacteria select when TCA cycle is non-functional. EU genes included the regulator of glutamine synthase (Rv2221c) suggesting a possible synthesis of alpha-ketoglutarate to feed TCA through that component, or gene *dct*A putatively involved in the transport of dicarboxylates, such as succinate, fumarate, and malate, across the membrane. Finally, some genes related to TCA cycle pathway showed responses to growth arrest that did not appear significantly affected by iron (genes outside the EU or ED sets, controlling reactions represented by brown arrows in Figure 6) suggesting a putative function in the survival of Mtb in the conditions tested.

Another important metabolic route that appears to be more downregulated upon growth arrest in the absence of iron was oxidative phosphorylation. Up to 13 of 14 genes coding for NADH dehydrogenase were ED genes (Supplementary Table 4) showing that, under iron deprivation, this main electron-transfer mechanism is more strongly repressed (Kurthkoti et al., 2017). Other ED genes included regulators of the synthesis of cytochrome C (Rv0527 and Rv0529) also members of the canonical oxidative phosphorylation route. Searching for an alternative to electron transport ways, we found that four cytochrome P450 genes (*cyp*51, *cyp*123, *cyp*138, and *cyp*142) and the ferredoxin reductase FprB were EU genes (Supplementary Table 4). All the mycobacterial cytochrome P450 are dependent on NAD(P)H ferredoxin reductase FprB, whose activity generates NADPH, as the source of precursors of bioenergetic products (Ortega Ugalde et al., 2019). Therefore, the blockage found for NADH dehydrogenase could be replaced by the cytochrome P450 and FprB system in the generation of energy sources.

The growth arrest derived from the establishment of stationary phase has an important influence on ribosome synthesis and its functional activity, since the bacteria do not just slow down cell division but also should slow down protein synthesis, through regulatory mechanisms involving certain toxin/antitoxin (T/A) pairs and ncRNAs (Sala et al., 2014). One example of these regulatory T/As is the T/A system *maz*E6*/*F6, which is involved in the cleavage of the 23S rRNA from mature ribosomes with the resulting decline of protein synthesis (Sala et al., 2014). Importantly, this T/A was an EU gene in our data (Supplementary Table 4). Furthermore, several stable RNAs were also found within the EU-set. They include 23S rRNA (*rrl*), the RNA subunit of RNase P (*rnp*B) that participates in the synthesis of mature-tRNA (Herrmann et al., 2014), as well as 10S RNA (*ssr*) the only tmRNA identified in the Mtb genome. The tmRNA performs the trans-translation reaction, making possible the rescue of ribosomes by degrading stalled mRNA during interrupted translation, suggesting a relationship with the bacterial response to ribosome-inhibiting conditions (Andini and Nash, 2011). Functional *ssr* requires the junction with protein SmpB (Personne and Parish, 2014), the corresponding gene coding for this protein (*smp*B) does not have a significant DE at iron-growth interaction in our data (Supplementary Table 4).

### Lipids characterization

**FIGURE 7:**
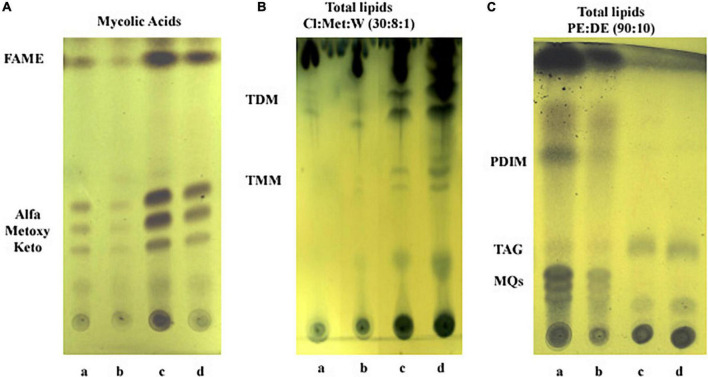
Analysis of lipid content by thin layer chromatography. **(A)** Mycolic acids. FAME, fatty acids methyl esters. **(B)** Polar lipids: TDM, trehalose dimycolate; TMM, trehalose monomycolate; Cl, chloroform; Met, methanol; W, water. **(C)** Apolar lipids: PDIM, phthiocerol dymycocerosate; TAG, triacylglycerol; MQs, menaquinones; PE, petroleum ether; DE, diethyl ether. Lanes: a, Exp5–Fe; b, Exp5+Fe; c, Stat6–Fe; and d, Stat6+Fe.

To gain insight into lipid changes linked to the effect of iron and growth arrest, TLC analysis was performed on whole Mtb cells submitted to the four different conditions under study: Exp5 and Stat6, any of them; with (+Fe) and without iron (−Fe). No differences were detected in the mycolic acid composition of the bacteria under the different conditions used (Figure 7A). Concerning total lipid analysis, conditions to develop polar and non-polar lipids were applied (Figures 7B,C). The analysis of polar lipids showed a higher abundance of trehalose mono- (TMM) and dimycolates (TDM) at Stat6 phase compared to Exp5 phase (Figure 7B). The opposite result was observed when apolar lipids were analyzed (Figure 7C). Further characterization to confirm the detection of PIMs in Exp5 phase, was performed by using two-dimensional TLC (Supplementary Figure 6). Interestingly, by applying conditions aimed at resolving apolar lipids, we observed that the band corresponding to PDIM was visible at Exp5 phase but was not detected at Stat6 phase independently of the iron content (Figure 7C). Similar to previous data (Bacon et al., 2007) increased levels of MQs were detected in iron starvation during exponential phase (Figure 7C). We also detected increased levels of TAG in stationary phase, in agreement with the detected higher proportion of red-nile stained bacilli (Figure 2).

## Discussion

Soon after entering inside its primary cellular target in the host –the human macrophage–, Mtb needs to adapt to an intracellular environment to establish infection. In this niche, bacteria undergo a set of metabolic changes that allow their survival and persistence within the phagosome. Those changes include slowing down both replication and metabolism, which eventually leads to establishing a successful long-term latent infection (Henry Boom et al., 2021). Complementarily, these also imply the deployment of specific transcriptional programs devoted to counteract the harmful effects of the multiple stressors that characterize the intracellular milieu (Schnappinger et al., 2003). Arguably, among these stressful cues, one of the most relevant is the reduced availability of key nutrients such as iron (Chao et al., 2019). In response to the low iron levels found in the phagosome, Mtb activates a series of molecular pathways aiming at securing the access, internalization and storage of this key nutrient, either in its free form, or bound to host’s heme molecules (Chao et al., 2019; Zhang et al., 2020); a result that we have replicated in our data in both exponential and stationary phases. In spite of how widely acknowledged it is the importance of activating these homeostasis mechanisms in response to iron deprivation, before this study it was unclear how the effects of iron deprivation on gene regulatory programs depend upon the growth phase of the bacteria or whether they affect, or not, the transcriptional response to growth arrest itself, and, by all means, in which direction.

To shed light on this specific question, we have conducted transcriptomic and lipidomic experiments in a factorial design, which allowed us to evaluate how the transcriptomic effects of iron availability depend on the growth phase.

In our data, iron deprivation was followed by the activation of mycobactin genes, both at the Exp5 and Stat6 phases. However, a novel result enabled by our factorial design is the remarkably asymmetric character of the effects of iron in relation to the growth phase, since, beyond the mentioned mycobactin genes (*n* = 8, highlighted in green in Supplementary Figure 3), none of these iron deprivation effects on gene expression found at the stationary phase were replicated under exponential growth (Figure 3B) with enough statistical significance. Surprisingly, even if Mtb is thought to recur to sequestrating host iron through heme-dependent mechanisms when growing extracellularly [e.g., in the bloodstream or within blood-rich cavitary lesions during active TB (Chao et al., 2019)] more intensely than under dormancy, in our data, genes controlling heme-depending iron sequestration and homeostasis were not activated upon iron deprivation in the exponential phase.

The lack of signal for iron effects at exponential phase seems to be in conflict with microarrays results previously reported in Rodriguez et al. (2002), where 285 genes were reported to be DE in response to iron deprivation in exponential cultures. However divergent the sensitivity of our statistical models seem to be at capturing iron effects at exponential growth, fold change effects reported by Rodriguez et al. (2002) appear significantly correlated to ours (*r* = 0.49, *p* = 8.4E−18, Supplementary Figure 7A), and the set of DE genes reported in their study appear enriched at the top of the rank for the genes with a greater evidence of iron dependency in our data as determined by a gene set enrichment analysis (Korotkevich et al., 2021) (*p* = 1.3E−11, Supplementary Figure 7B). These comparative metrics suggest a high degree of correlation between results reported by Rodriguez et al. (2002) and our analyses, which turns out to be more conservative at assigning statistical significance. The differences between both studies may arise both from technical sources that limit replicability (e.g., microarrays versus RNA-seq), as well as from biological aspects, among which, the carbon source used was different in each study: glucose + glycerol (Rodriguez et al., 2002) versus only glycerol (this study). Be it as it may, our analyses lead to the identification of a stronger signal for iron effects at the stationary phase (797 DE genes at FDR < 0.01) than what was found at the exponential phase either in this study, or in Rodriguez et al. (2002), where iron effects at the stationary phase were not analyzed.

The factorial nature of our experimental design unlocks the interrogation of interaction effects between iron availability and growth phase, namely, the identification of genes whose transcriptional adaptation to growth arrest is modulated by iron availability. Surprisingly, among the genes for which the size of the transcriptomic responses to growth arrest were found to be iron dependent, as many as 81.2% (429 over 528 genes), deployed stronger responses to growth arrest under iron deprived conditions, suggesting a global role for iron deprivation as a factor contributing to a deeper transcriptional shift towards slow replication.

As expected, among genes whose upregulation upon growth arrest is enhanced by iron starvation (EU genes), we found a battery of genes enabling sequestration, internalization and storage of iron, recapitulating findings previously described in the literature. Deprivation of iron, mainly under growth arrest, derived in overexpression of IdeR, the central transcriptional regulator of iron. IdeR regulon contains the two loci of mycobactins (*mbt* 1 and 2) and the two main iron storage proteins (bacterioferritin BbfrA and ferritin-like protein BbfrB) (Gold et al., 2001; Choudhury et al., 2021). Therefore, also in our data, this overexpression was followed by upregulation of mycobactins and bacterioferritin BfrA, together with a whole set of genes participating in iron homeostasis (Supplementary Figure 5). Gold et al. (2001) described that *bfr*A was under the control of two promoters, each of them differentially used according to iron availability. Under low iron conditions, the low-iron promoter is preferentially used. On the contrary, BfrB showed greater capacity for iron storage in high-iron environments (Khare et al., 2017). In accordance to these previous data we found enhanced downregulation of *bfr*B upon growth arrest in iron deprived condition (Supplementary Figure 5).

Furthermore, we observed how iron deprivation enhances transcriptional responses related to other environmental cues that are in themselves independent to iron (Figure 4B). This includes responses to oxygen reactive species, starvation of other nutrients further than iron, host immune responses and sterol exposures, as well as exposure to toxic metal ions.

Metals, in trace amounts, are essential for the survival of bacteria, even though they are also toxic when present in excess (Marcela Rodriguez and Neyrolles, 2014). This fact is used by the macrophage, that tries to intoxicate the bacteria with exposing it to high levels toxic of metals such as copper (Cu^+^/Cu^2+^) or zinc (Zn^2+^) that are harmful to the bacterium. In other occasions (as it happens with Mn^2+^), the host synthesizes proteins that sequester some of these metals to impair pathogen’s access to these key nutrients thus limiting the bacterial growth. All these processes constitute the human homeostatic responses that eventually control the disease (Cassat and Skaar, 2013), which are in turn counteracted by the bacterium through a series of molecular strategies. Importantly, our results showed for the first time that iron deprivation serves as a generic cue after which the bacterium enhances the upregulation of many of the genes involved in ensuring the homeostasis of other metals that include copper, zinc, cadmium, and nickel, among others (Figure 5).

We also found that several ncRNAs behave as EU genes in our conditions. This is expected, considering that ncRNAs seem to play an important role for Mtb when bacilli slow down cell division (Ignatov et al., 2015). Up to three of those ncRNAs detected were involved in the control of ribosome functionality. Moreover, we also detected upregulation of *car*D gene (Rv3583) in response to growth arrest (Supplementary Table 4). This last gene seems to be essential for rRNA synthesis in mycobacteria, mainly under stringent conditions (Stallings et al., 2009). Therefore, Mtb modifies their transcriptomic responses to maintain a basal ribosomal functionality, as relevant survival requirement.

While we found that iron enhances the activation of response mechanisms to intracellular stressors in the transition to stationary phase, our analyses equally indicated that the main genes and pathways whose activity is repressed upon growth arrest are more intensely blocked in low-iron cultures. This “enhanced downregulation” profile (ED genes) impacts primarily genes in the TCA cycle and oxidative phosphorylation pathways, suggesting that lack of iron intensifies the adaptation of Mtb to conditions that required metabolic switch to non-canonical routes for carbon usage and energy production, similarly to that is observed upon entering into dormancy. This is in agreement with previously published microarray studies reporting gene expression changes in iron deprived bacteria (Kurthkoti et al., 2017) and in starvation (Betts et al., 2002). In spite of this general pattern, some of the components of the TCA cycle appear upregulated in the transition to the stationary phase, even more in −Fe cultures, including *icl*1, the primary step in the glyoxylate shunt, suggesting that a basic minimal carbon metabolic activity of TCA cycle remains necessary regardless iron availability or even growth phase.

The cell wall of Mtb contains a relevant number of different lipids that participate in the pathogenesis of the disease. However, the influence of iron availability on lipid content and composition of the bacilli has received little attention. In our TLC experiments, we did not find robust evidence of significant changes in mycolic acid and polar lipid either as a function of the growth phase or iron availability levels, as summarized in Figures 7A,B. On the contrary, the important lipid PDIM was not detected in Stat6, even this effect was similarly observed in +Fe and −Fe cultures. Domenech and Reed (2009) described that H37Rv could spontaneously lose its capacity to synthesize PDIM after several *in vitro* subcultures, leading to an attenuated phenotype. This last consideration does not occur in our case, due to detection of PDIM in exponential cultures (Figure 7C). To determine if gene expression could be related to the absence of PDIM in Stat6, we checked the transcriptomic responses to growth arrest of genes involved in the synthesis and transport of PDIM (Rens et al., 2021; Supplementary Table 4). No DE was observed in one third (8/26) of the genes associated with PDIM synthesis. A single gene, *fad*D26, was upregulated, while half of the genes (12/26) were downregulated in stationary phase regardless iron availability. Genes downregulated included all the four genes related to transport and localization of PDIM in the cell envelope (FDR = 3.3E−11 to 3.1E−29; mean value 4.17E−12) and half of the genes related to PDIM synthesis (5/11) (FDR = 7.1E−04 to 1.3E−134; mean value 8.1E−12). Kurthkoti et al. (2017) detected downregulation of several *fad*D genes involved in the synthesis of PDIM, including *fad*D26, in iron deprived cultures. The different culture media applied could explain that difference, compared to our results. PDIM is considered a virulent factor of Mtb because it is required for the survival of the bacilli inside the phagosome (Rens et al., 2021). Moreover, it has been associated with propionyl-CoA detoxification, derived from metabolism of cholesterol and other odd fatty acids (Singh et al., 2009). Detoxification of propionyl-CoA might occur either using the methylcitrate or the methylmalonate pathways (Queiroz and Riley, 2017). In our conditions, genes involved in both metabolic routes were down regulated irrespective of iron availability (Supplementary Table 4) discarding the functionality of those routes. Our data showed that PDIM decreased its level in conditions where the bacilli enter into a silent, slow dividing phenotype, most probably similarly to that in the human host when establish long-term latent infection, a situation in which virulent factors are, most probably, not required.

## Conclusion

Our results highlight a significantly weaker influence of iron deprivation on the transcriptional profile of Mtb at the exponential phase than at the stationary phase of growth. This asymmetry translates into the presence of significant effects of iron levels on responses to growth arrest, that have been characterized in detail for the first time in this study.

Taken together, the results of these analyses suggest a role for iron deprivation as a signaling cue that primes the bacterium to respond more strongly to growth arrest towards the silent phenotype. According to this view, bacteria undergo amplified transcriptomic responses when entering into a dormant-like phenotype in the lack of iron. As we have seen through our analyses, this results in the enhanced activation of more intense positive responses to several intracellular stresses, such as metals deregulation, oxidative stress, or nutrient starvation; as well as in the induction of a more stringent shutdown of cellular respiration, and central carbon metabolic pathways, and ribosomal activity. From an evolutionary perspective, this suggests that Mtb, as an intracellular obligate parasite, has evolved to recognize the multi-dimensional threads posed by the hostile intracellular environment, and respond to all of them coherently, using iron levels as a general signaling proxy of overall stress to quantitatively modulate the intensity of the dormancy transcriptional shift.

In spite of the functional significance of the effects of iron on the transcriptional responses to growth arrest, lipid composition remained comparably less affected in our cultures, either by the growth profile, but specially by the disparate iron levels scrutinized. While the major virulence lipids family of PDIMs was only found at the exponential phase, its abrogation upon growth arrest was found to be independent of iron availability, in contrast to the transcriptional differences described.

Disentangling more in-depth these and other adaptation mechanisms deployed by the bacteria in its transition between replicative and dormant states is crucial for the development of novel prophylactic and bactericidal drugs, and deployment of effective control measures for Tuberculosis.

## Data availability statement

The data presented in this study are deposited in the Gene Expression omnibus repository, accession number: GSE213943.

## Author contributions

MJG, MCM, and JS conceived and designed the study. SA, MJG, and MCM conducted the experiments. SA, JS, JC-P, and LV conducted the bioinformatic work flow. MJG, MCM, JS, JC-P, and PDP performed the data interpretation. MJG, MCM, JS, RP-R, and PDP wrote and revised manuscript. All authors approved the manuscript.

## Supplementary material

Supplementary Figure 1 Number of DE genes in response to growth arrest in +Fe (blue) and −Fe (orange) cultures. Out of 2,725 genes present in the union of both sets, 1,903 show growth arrest effects that are shared regardless iron.

Supplementary Figure 2 Distributions of logFC responses to growth arrest in −Fe (top) and +Fe cultures (bottom), split by feature biotype (protein coding versus ncRNAs). Effect size distributions of non-protein coding genetic features are shifted towards positive, larger values with respect to protein coding genes [Mann–Whitney tests *p* = 2.2E−10 (at Fe/+), and *p* = 2.8E−11 (at −Fe)].

Supplementary Figure 3 Fold change responses to iron deprivation of genes coding for mycobactins at Exp5 and Stat6 phases of growth.

Supplementary Figure 4 Heat map with expression levels of 429 genes showing increased response to growth arrest under iron deprivation [interaction, contrast (v)]. Values of both replicates were normalized log2 cpm (counts per million) expression patterns for genes in the EU (enhanced upregulated, left panel) and ED (enhanced downregulated, right panels), mean centered within each iron level. See text for more explanation.

Supplementary Figure 5 Genes related to iron homeostasis. Circular red and green signals indicate, respectively, the repression or activation of the corresponding regulatory gene-target in response to growth arrest being enhanced by iron deprivation [interaction, contrast (v)]. Light oval gray, regulator-related promoter region; dark oval gray, metal-repressor complex related promoter region; small red circles, iron metal. Blue: downregulated response to interaction (ED genes). Black: upregulated responses to interaction (EU genes). See Supplementary Table 4 for gene expression levels.

Supplementary Figure 6 Analysis of PIMs content. Phosphatidyl-inositol mannosides content by two dimensional thin layer chromatography. **(A)** Lipid extraction from exponential phase with (+Fe) and without (−Fe) iron. **(B)** Lipid extraction from stationary phase with (+Fe) and without (−Fe) iron. PI, phosphatidyl-inositol; Ac, acetylated residues; M, mannosides residues.

Supplementary Figure 7 Iron deprivation effects at Exp5 compared to effects reported in Rodriguez et al. (2002). **(A)** Scatter plot comparing the log2 FC reported by Rodriguez et al. (2002) (*x*-axis; iron deprived minus iron rich) for the genes labeled as iron dependent at exponential phase in their study versus the raw log2FC estimated by DEseq2 in our analyses. In turquoise, genes labeled as DE in our data, which share simultaneously the largest effect sizes in both studies. **(B)** Gene set enrichment analysis plot, obtained using FGSEA (Korotkevich et al., 2021) indicating a strong enrichment (*p* = 1.3E−11) of genes labeled as iron dependent by Rodriguez et al. (2002) among the genes showing most compelling evidence of differential expression in our data (largest absolute values of raw log2FCs divided by standard errors ratios).
