# Corrigendum: Iron deprivation enhances transcriptional responses to *in vitro* growth arrest of *Mycobacterium tuberculosis*

**DOI:** 10.3389/fmicb.2022.1081051

**Published:** 2022-11-29

**Authors:** Sogol Alebouyeh, Jorge A. Cárdenas-Pestana, Lucia Vazquez, Rafael Prados-Rosales, Patricia Del Portillo, Joaquín Sanz, Maria Carmen Menéndez, Maria J. García

**Affiliations:** ^1^Department of Preventive Medicine and Public Health and Microbiology, School of Medicine, Autonomous University of Madrid, Madrid, Spain; ^2^Department of Theoretical Physics, University of Zaragoza, Zaragoza, Spain; ^3^Institute for Biocomputation and Physics of Complex Systems (BIFI), University of Zaragoza, Zaragoza, Spain; ^4^Corporación CorpoGen, Bogota, Colombia

**Keywords:** *Mycobacterium tuberculosis*, iron availability, transcriptomics, growth arrest, metabolic changes

In the published article, there was an error in the legend of Figure 7B as published.

Analysis of lipid content by thin layer chromatography. **(A)** Mycolic acids. FAME, fatty acids methyl esters. **(B)** Polar lipids: PGL, phenolic glycolipid; GPL, glycopeptidolipids; TMM, trehalose monomycolate; PIMs, phosphatidyl-inositol mannosides; Cl, chloroform; Met, methanol; W, water. **(C)** Apolar lipids: PDIM, phthiocerol dymycocerosate; TAG, triacylglycerol; MQs, menaquinones; PE, petroleum ether; DE, diethyl ether. Lanes: a, Exp5–Fe; b, Exp5+Fe; c, Stat6–Fe; and d, Stat6+Fe.

The corrected legend appears below.

In the published article, there was an error in Figure 7B as published. Names of lipids are wrongly label.

The corrected Figure 7 and its caption appear below.

**Figure 7:**
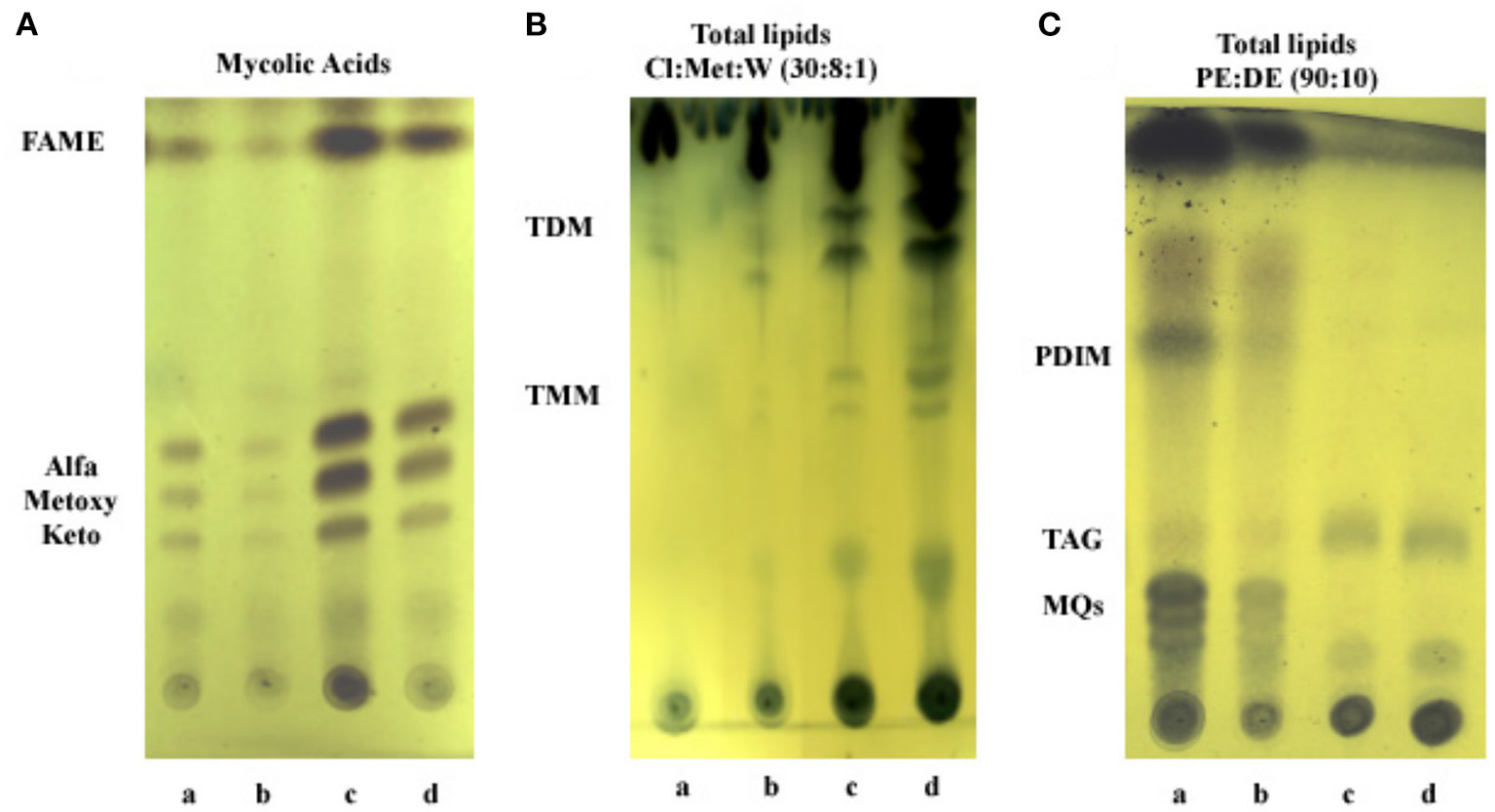
Analysis of lipid content by thin layer chromatography. **(A)** Mycolic acids. FAME, fatty acids methyl esters. **(B)** Polar lipids: TDM, trehalose dimycolate; TMM, trehalose monomycolate; Cl, chloroform; Met, methanol; W, water. **(C)** Apolar lipids: PDIM, phthiocerol dymycocerosate; TAG, triacylglycerol; MQs, menaquinones; PE, petroleum ether; DE, diethyl ether. Lanes: a, Exp5–Fe; b, Exp5+Fe; c, Stat6–Fe; and d, Stat6+Fe.

In the published article, there was an error. Page 10, **Results**.

A correction has been made to section **Results**, subsection **Lipids characterization**. This sentence previously stated:

“To gain insight into lipid changes linked to the effect of iron and growth arrest, TLC analysis was performed on whole Mtb cells submitted to the four different conditions under study: Exp5 and Stat6, any of them; with (+Fe) and without iron (−Fe). No differences were detected in the mycolic acid composition of the bacteria under the different conditions used (Figure 7A). Concerning total lipid analysis, conditions to develop polar and non-polar lipids were applied (Figures 7B,C). The analysis of polar lipids showed a higher abundance of PIMs and glycopeptidolipids (GPL) at Stat6 phase compared to Exp5 phase (Figure 7B). The opposite result was observed when apolar lipids were analyzed (Figure 7C). Further characterization to confirm the detection of PIMs in Exp5 phase, was performed by using two-dimensional TLC (Supplementary Figure 6). Interestingly, by applying conditions aimed at resolving apolar lipids, we observed that the band corresponding to PDIM was visible at Exp5 phase but was not detected at Stat6 phase independently of the iron content (Figure 7C). Similar to previous data (Bacon et al., 2007) increased levels of MQs were detected in iron starvation during exponential phase (Figure 7C). We also detected increased levels of TAG in stationary phase, in agreement with the detected higher proportion of red-nile stained bacilli (Figure 2).”

The corrected sentence appears below:

“To gain insight into lipid changes linked to the effect of iron and growth arrest, TLC analysis was performed on whole Mtb cells submitted to the four different conditions under study: Exp5 and Stat6, any of them; with (+Fe) and without iron (-Fe). No differences were detected in the mycolic acid composition of the bacteria under the different conditions used (Figure 7A). Concerning total lipid analysis, conditions to develop polar and non-polar lipids were applied (Figures 7B,C). The analysis of polar lipids showed a higher abundance of trehalose mono- (TMM) and dimycolates (TDM) at Stat6 phase compared to Exp5 phase (Figure 7B). The opposite result was observed when apolar lipids were analyzed (Figure 7C). Further characterization to confirm the detection of PIMs in Exp5 phase, was performed by using two-dimensional TLC (Supplementary Figure 6). Interestingly, by applying conditions aimed at resolving apolar lipids, we observed that the band corresponding to PDIM was visible at Exp5 phase but was not detected at Stat6 phase independently of the iron content (Figure 7C). Similar to previous data (Bacon et al., 2007) increased levels of MQs were detected in iron starvation during exponential phase (Figure 7C). We also detected increased levels of TAG in stationary phase, in agreement with the detected higher proportion of red-nile stained bacilli (Figure 2).”

The authors apologize for this error and state that this does not change the scientific conclusions of the article in any way. The original article has been updated.

## Publisher's note

All claims expressed in this article are solely those of the authors and do not necessarily represent those of their affiliated organizations, or those of the publisher, the editors and the reviewers. Any product that may be evaluated in this article, or claim that may be made by its manufacturer, is not guaranteed or endorsed by the publisher.
