# Correction: Iron deprivation enhances transcriptional responses to *in vitro* growth arrest of *Mycobacterium tuberculosis*

**DOI:** 10.3389/fmicb.2025.1699855

**Published:** 2025-09-26

**Authors:** Sogol Alebouyeh, Jorge A. Cárdenas-Pestana, Lucia Vazquez, Rafael Prados-Rosales, Patricia Del Portillo, Joaquín Sanz, Maria Carmen Menéndez, Maria J. García

**Affiliations:** ^1^Department of Preventive Medicine and Public Health and Microbiology, School of Medicine, Autonomous University of Madrid, Madrid, Spain; ^2^Department of Theoretical Physics, University of Zaragoza, Zaragoza, Spain; ^3^Institute for Biocomputation and Physics of Complex Systems (BIFI), University of Zaragoza, Zaragoza, Spain; ^4^Corporación CorpoGen, Bogota, Colombia

**Keywords:** *Mycobacterium tuberculosis*, iron availability, transcriptomics, growth arrest, metabolic changes

An incorrect **Funding** statement was provided. The correct funder is “ISCIII”. The correct funding statement reads: “MJG and MCM acknowledged the support from FEDER (a way to construct Europe) PI19/00666, ISCIII. RP-R acknowledged the support from NIH RO1AI162821 and support by MICINN contract PID2019-110240RB-I00. JS and JC-P acknowledged support from grants PID2019-106859GA-I00 and RYC-2017-23560 funded by MCIN/AEI/10.13039/501100011033 and by ESF Investing in your future. JS acknowledged partial support from the Government of Aragon through grant E36-20R (FENOL).”

The original version of this article has been updated.

