# Supplementary material for: Iron deprivation enhances transcriptional responses to in vitro growth arrest of Mycobacterium tuberculosis
Source: Front Microbiol. 2022 Oct 4;13:956602. doi: 10.3389/fmicb.2022.956602 (PMC9577196; doi:10.3389/fmicb.2022.956602)
Supplement: Supplementary file 1 [file Data_Sheet_1.PDF]

Supplementary Tables 1 & 2

Table S1. **RNA quality control and quantification**.

Cultures	RNA (ng/µl)	260/280	260/230	RIN	rRNA ratio (23S/16S)
Exp5-Fe (1)	616	1.9	2.1	7.2	0.8
Exp5-Fe (2)	405	2.0	2.0	6.9	0.5
Exp5+Fe (1)	459	1.9	2.1	7.4	0.6
Exp5+Fe (2)	454	2.0	2.0	9.4	0.4
Stat6-Fe (1)	602	1.9	2.1	8.4	0.5
Stat6-Fe (2)	648	1.9	2.1	8.4	0.6
Stat6+Fe (1)	692	1.9	2.2	8	0.7
Stat6+Fe (2)	1023	1.9	2.1	8.6	0.6

Number of the replicas indicated between brackets.
Exp5, exponential cultures; Stat6, stationary cultures; -Fe, cultures without iron; +Fe, cultures with iron.

Table S2. **Lipids isolation and quantification**.

Cultures	Mycolic acids	Total lipids
Exp5-Fe (1)	nd	nd
Exp5-Fe (2)	2.4mg	48µl
Exp5+Fe (1)	4.1mg	82µl
Exp5+Fe (2)	3.8mg	76µl
Stat6-Fe (1)	3.1mg	62µl
Stat6-Fe (2)	nd	nd
Stat6+Fe (1)	4.2mg	84µl
Stat6+Fe (2)	2.8mg	56µl

Amount of mycolic acids isolated (left column) and volumen of CH_2CL_2 added to the total lipids extracts (right column) for 50µg/µl as final concentration. Number of the replica indicated between brackets.
Exp5, exponential culture; Stat6, stationary culture; -Fe, cultures without iron; +Fe, cultures with iron; nd, no data.
