# Supplementary figures and images for: Iron deprivation enhances transcriptional responses to in vitro growth arrest of Mycobacterium tuberculosis
Source: Front Microbiol. 2022 Oct 4;13:956602. doi: 10.3389/fmicb.2022.956602 (PMC9577196; doi:10.3389/fmicb.2022.956602)

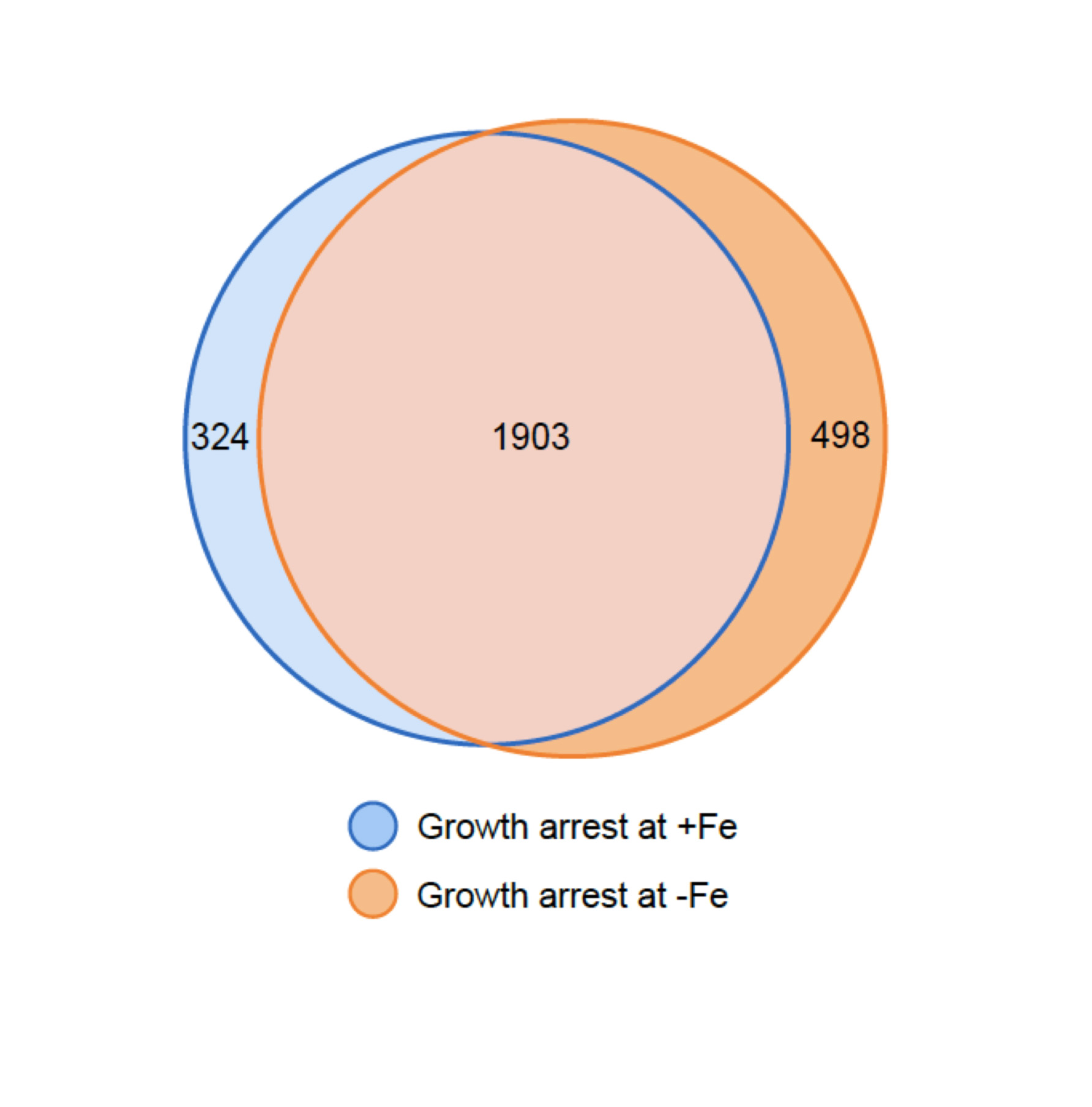

Supplement: Supplementary Figure 1 — Number of DE genes in response to growth arrest in +Fe (blue) and −Fe (orange) cultures. Out of 2,725 genes present in the union of both sets, 1,903 show growth arrest effects that are shared regardless iron. [file Image_1.JPEG]

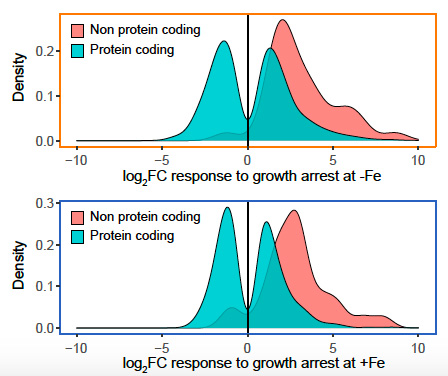

Supplement: Supplementary Figure 2 — Distributions of logFC responses to growth arrest in −Fe (top) and +Fe cultures (bottom), split by feature biotype (protein coding versus ncRNAs). Effect size distributions of non-protein coding genetic features are shifted towards positive, larger values with respect to protein coding genes [Mann–Whitney tests p = 2.2E−10 (at Fe/+), and p = 2.8E−11 (at −Fe)]. [file Image_2.JPEG]

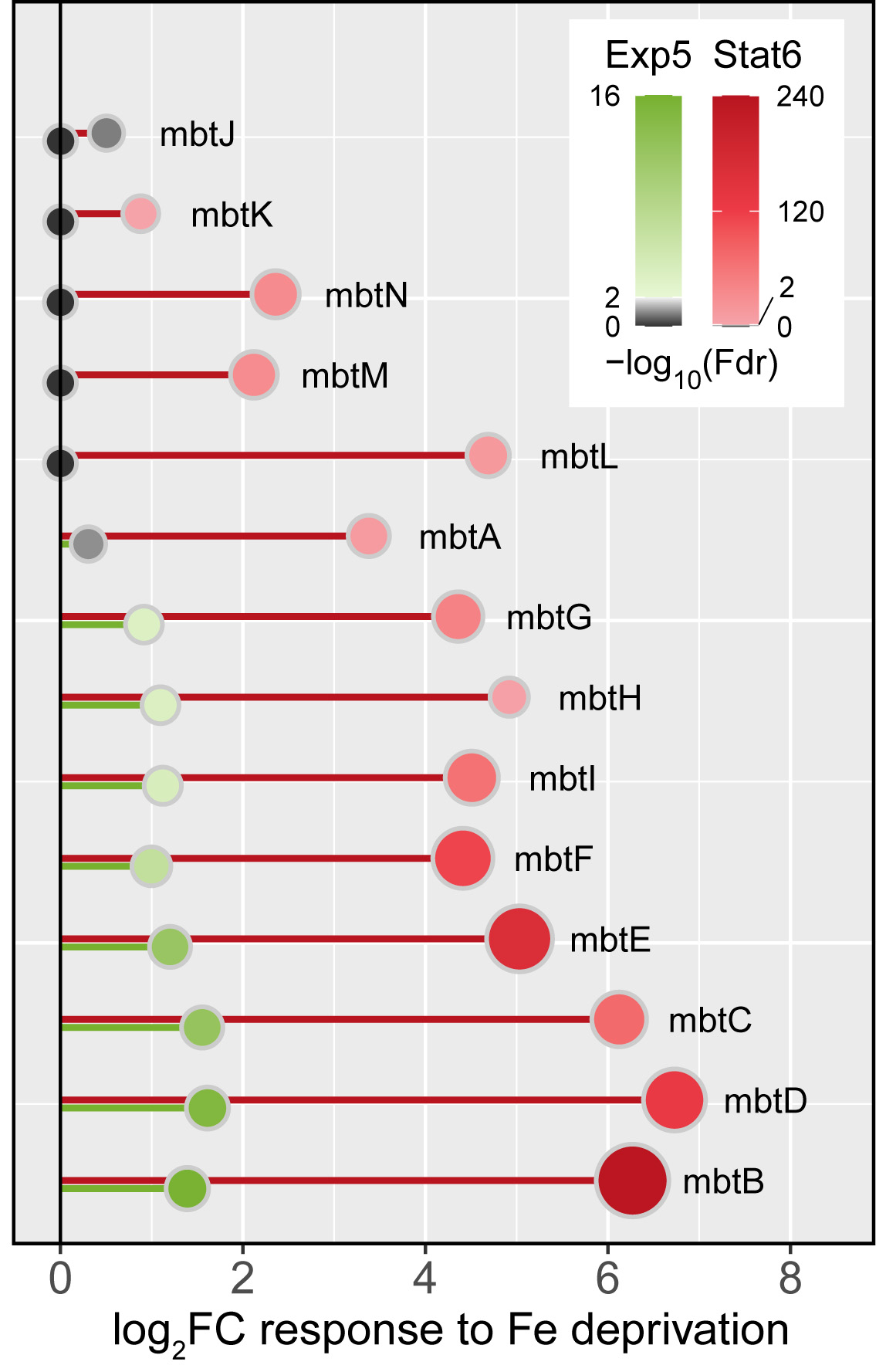

Supplement: Supplementary Figure 3 — Fold change responses to iron deprivation of genes coding for mycobactins at Exp5 and Stat6 phases of growth. [file Image_3.JPEG]

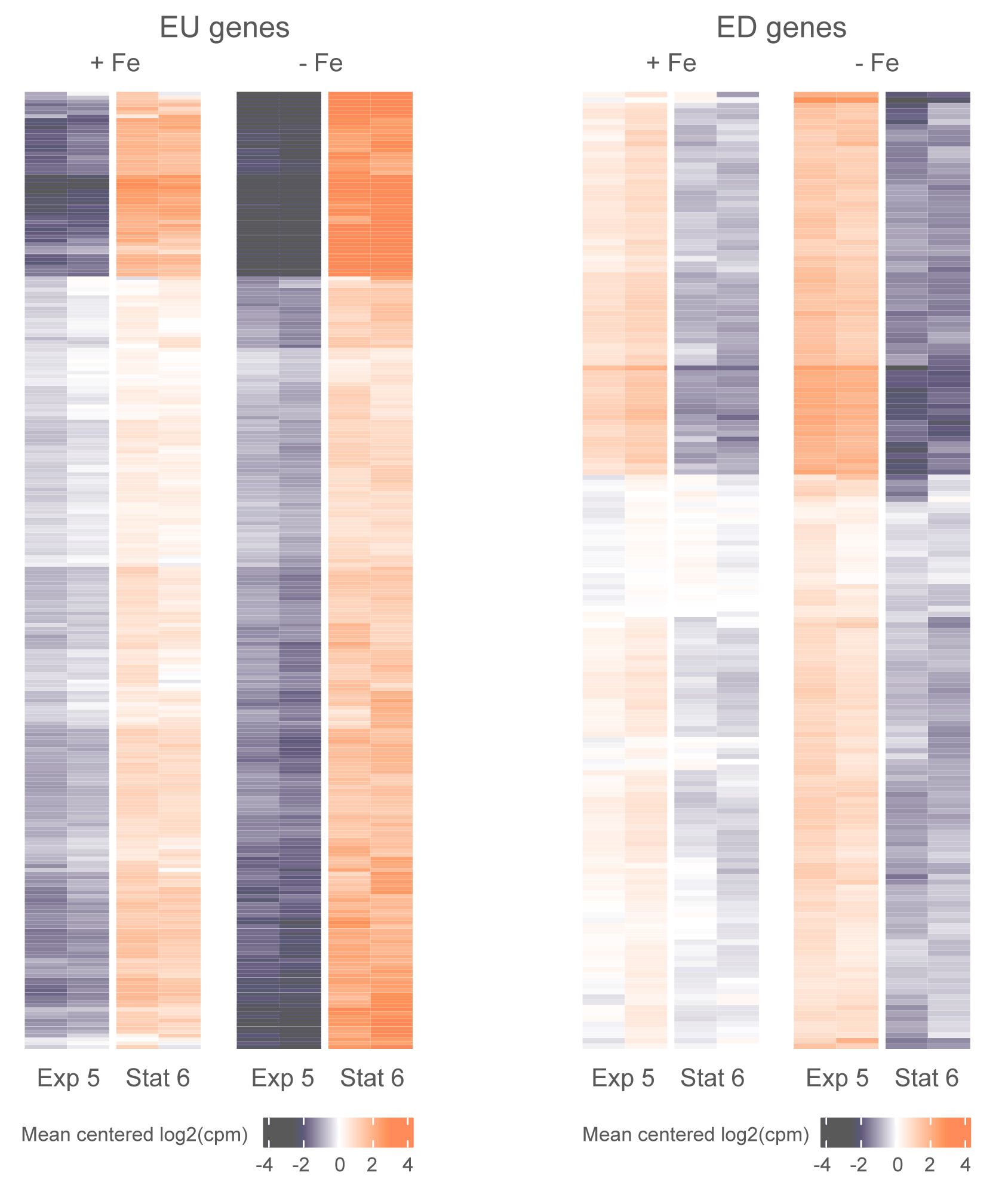

Supplement: Supplementary Figure 4 — Heat map with expression levels of 429 genes showing increased response to growth arrest under iron deprivation [interaction, contrast (v)]. Values of both replicates were normalized log2 cpm (counts per million) expression patterns for genes in the EU (enhanced upregulated, left panel) and ED (enhanced downregulated, right panels), mean centered within each iron level. See text for more explanation. [file Image_4.JPEG]

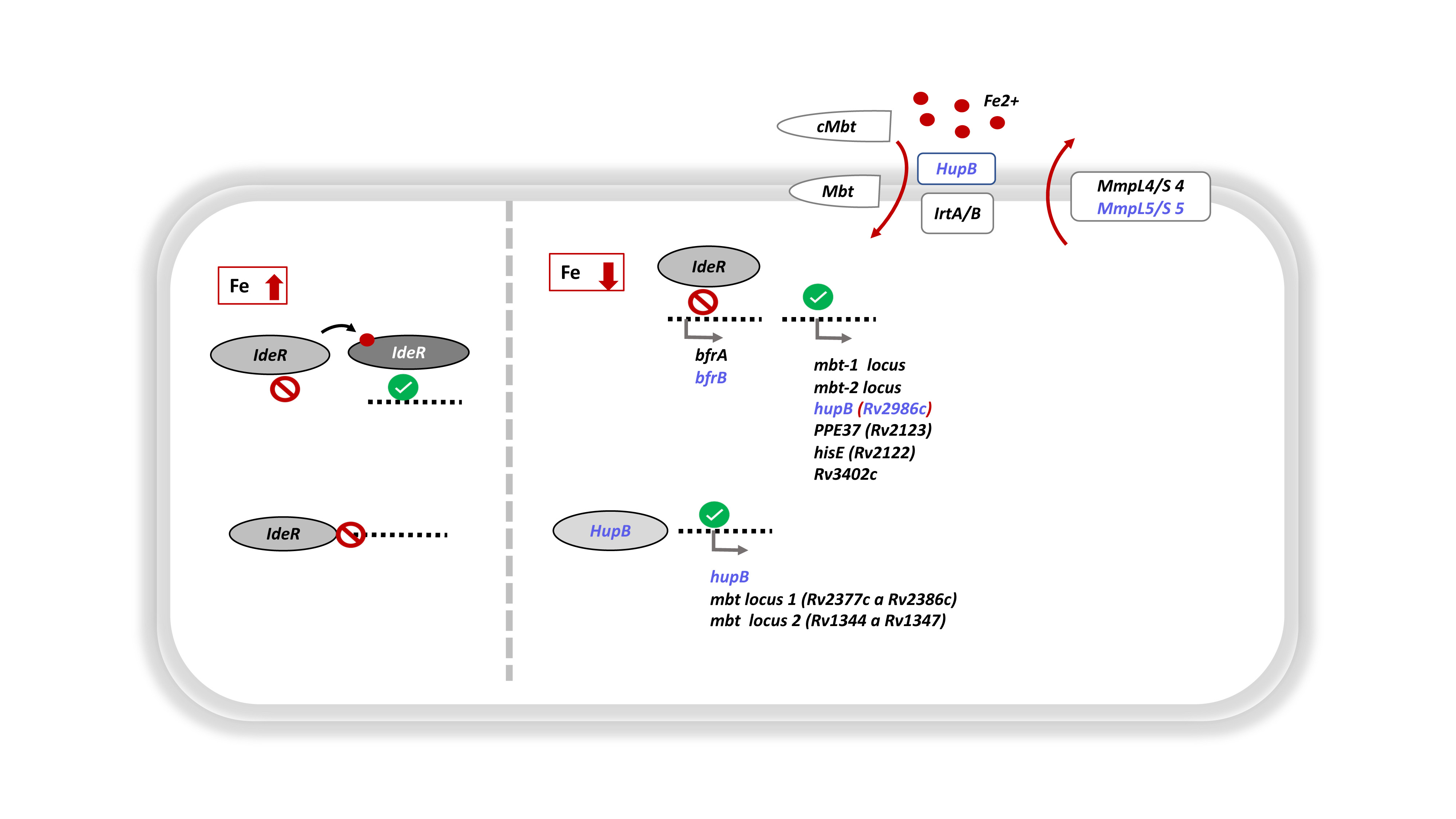

Supplement: Supplementary Figure 5 — Genes related to iron homeostasis. Circular red and green signals indicate, respectively, the repression or activation of the corresponding regulatory gene-target in response to growth arrest being enhanced by iron deprivation [interaction, contrast (v)]. Light oval gray, regulator-related promoter region; dark oval gray, metal-repressor complex related promoter region; small red circles, iron metal. Blue: downregulated response to interaction (ED genes). Black: upregulated responses to interaction (EU genes). See Supplementary Table 4 for gene expression levels. [file Image_5.JPEG]

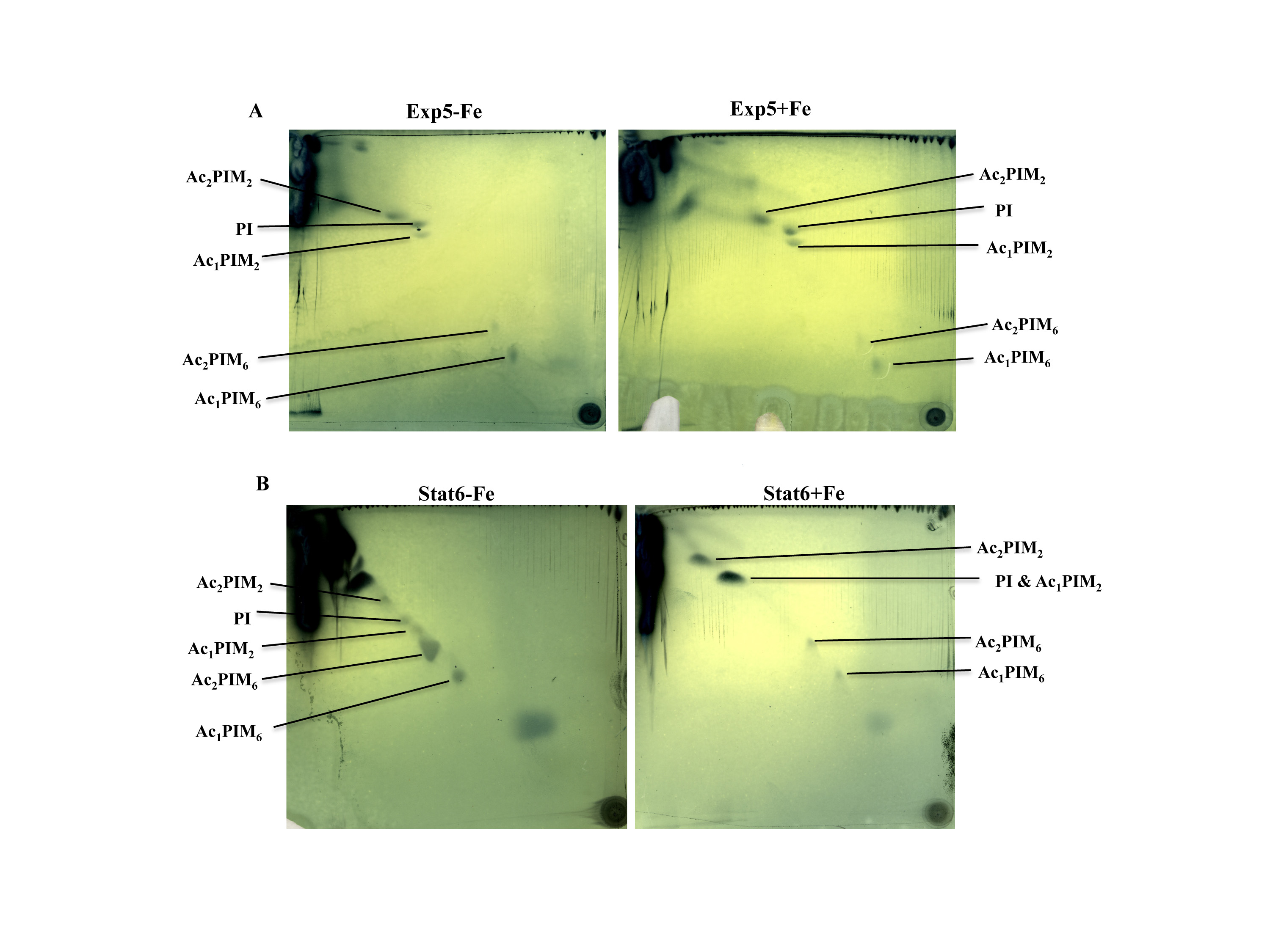

Supplement: Supplementary Figure 6 — Analysis of PIMs content. Phosphatidyl-inositol mannosides content by two dimensional thin layer chromatography. (A) Lipid extraction from exponential phase with (+Fe) and without (−Fe) iron. (B) Lipid extraction from stationary phase with (+Fe) and without (−Fe) iron. PI, phosphatidyl-inositol; Ac, acetylated residues; M, mannosides residues. [file Image_6.JPEG]

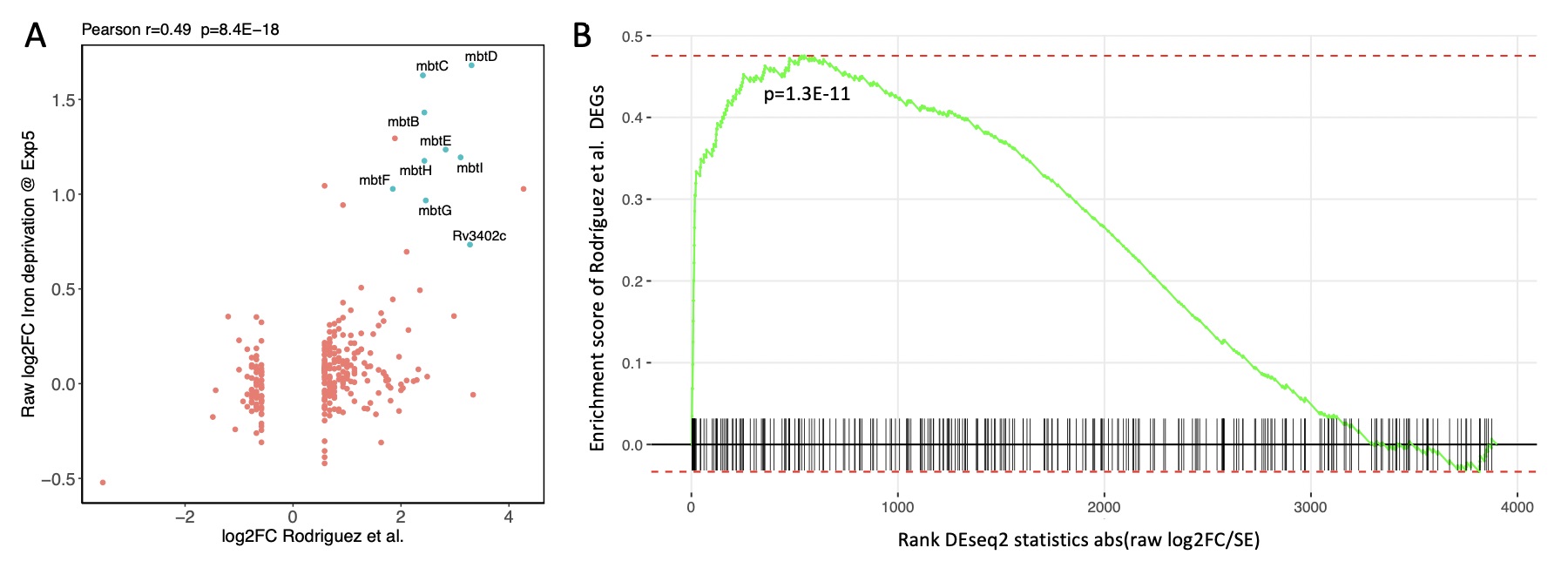

Supplement: Supplementary Figure 7 — Iron deprivation effects at Exp5 compared to effects reported in Rodriguez et al. (2002). (A) Scatter plot comparing the log2 FC reported by Rodriguez et al. (2002) (x-axis; iron deprived minus iron rich) for the genes labeled as iron dependent at exponential phase in their study versus the raw log2FC estimated by DEseq2 in our analyses. In turquoise, genes labeled as DE in our data, which share simultaneously the largest effect sizes in both studies. (B) Gene set enrichment analysis plot, obtained using FGSEA (Korotkevich et al., 2021) indicating a strong enrichment (p = 1.3E−11) of genes labeled as iron dependent by Rodriguez et al. (2002) among the genes showing most compelling evidence of differential expression in our data (largest absolute values of raw log2FCs divided by standard errors ratios). [file Image_7.JPEG]
